# The Design and Implementation of Cardiotocography Signals Classification Algorithm Based on Neural Network

**DOI:** 10.1155/2018/8568617

**Published:** 2018-12-03

**Authors:** Haijing Tang, Taoyi Wang, Mengke Li, Xu Yang

**Affiliations:** School of Computer Science and Technology, Beijing Institute of Technology, Beijing 10081, China

## Abstract

Mobile medical care is a hot issue in current medical research. Due to the inconvenience of going to hospital for fetal heart monitoring and the limited medical resources, real-time monitoring of fetal health on portable devices has become an urgent need for pregnant women, which helps to protect the health of the fetus in a more comprehensive manner and reduce the workload of doctors. For the feature acquisition of the fetal heart rate (FHR) signal, the traditional feature-based classification methods need to manually read the morphological features from the FHR curve, which is time-consuming and costly and has a certain degree of calibration bias. This paper proposes a classification method of the FHR signal based on neural networks, which can avoid manual feature acquisition and reduce the error caused by human factors. The algorithm will directly learn from the FHR data and truly realize the real-time diagnosis of FHR data. The convolution neural network classification method named “MKNet” and recurrent neural network named “MKRNN” are designed. The main contents of this paper include the preprocessing of the FHR signal, the training of the classification model, and the experiment evaluation. Finally, MKNet is proved to be the best algorithm for real-time FHR signal classification.

## 1. Introduction

With the general improvement of people's living standards in today's society, people's demands for health are constantly increasing, especially for the health of the next generation. More people want their children to be well cared for during their unborn childbirth. During the development of the fetus, parents hope to be able to grasp the physiological information of the fetus in real time, such as the fetal heart rate and contraction pressure, to help early detection of potential risks, and to guide fetal health development [1]. Due to psychological or physiological stress, the physiological parameters detected by some patients in the hospital and the physiological parameters detected in their familiar environment will be greatly different. Recent studies have shown that patients who are clinically diagnosed with high blood pressure and are likely to develop heart disease, after a 24-hour blood pressure monitoring at home, found that only one-third of the users' monitoring results are consistent with clinical diagnosis [2]. Professor Johnson has demonstrated the use of remote fetal monitoring methods to allow pregnant women and fetuses to measure important physiological indicators such as blood pressure, blood oxygen, and electrocardiogram at home [3]. This environment that allows pregnant women to feel at ease is more conducive to pregnant women, fetuses, and to detect the condition of the newborncorrectly [4]. Therefore, the use of remote fetal monitoring systems at home in perinatal pregnant women can improve the quality of perinatal care.

The purpose of this paper is to use neural network to classify the fetal heart rate monitoring data and realize real-time fetal health or abnormal development on the remote fetal heart monitoring system. Because the traditional FHR data classification uses complex professional features, these features require personnel with professional medical knowledge to accurately calibrate, and collection is slow and difficult [5]. In fact, the analysis of these FHR features is time consuming and laborious, and it is impossible to obtain medical conclusions in real time, which greatly delays the progress of medical diagnosis [6]. The latest neural network model can directly use the fetal heart rate data as input and automatically extract key features using relevant algorithms to obtain accurate classification results [7]. Therefore, we use neural network to explore the method of fetal heart monitoring data prediction. However, fetal heart rate monitoring data are faced with problems such as large data volume, high data noise, and serious data defects, which seriously affect the classification results. How to preprocess the collected raw data and design a neural network model that fits the data is the problem to be solved in this paper.

Based on the neural network algorithm, this paper design different FHR data classification models [8]. By comparing and evaluating the performance of different FHR data classification models, we summarize the performance of the above models and select the optimal FHR data classification model and finally use it to guide medical practice.

This paper will setup the control group and the experimental group. The control group adopts the traditional data classification methods, including the support vector machine (SVM) and the random forest (RF) method [9]. The experimental group adopts an artificial neural network (ANN) method, including convolution neural networks (CNN) and recurrent neural network (RNN) methods. These methods include the following: (1) data collection, (2) data preprocessing, (3) model training, and (4) evaluation.

## 2. Results

On the whole, the neural network-based training algorithm is superior to the feature-based training algorithm. As shown in Table 1, the length of the training time reflects the training speed of the classification model. By comparison, we can see that in the speed of the classification model, RF > SVM > MKNet (1330s = 19s∗70epoch) > MKRNN (350s = 5s∗70epoch). However, the length of training time is also related to the size of the data set and the complexity of the model [10]. In this paper, the simple comparison training time does not have great practical significance. Although the feature-based method is shorter in training time, the long manual marking time is also something we have to consider. In terms of accuracy, MKNet-C > MKRNN > RF > SVM.

The experimental results of different classification models will be described in detail below.

### 2.1. Support Vector Machine Evaluation

**Figure 1:**
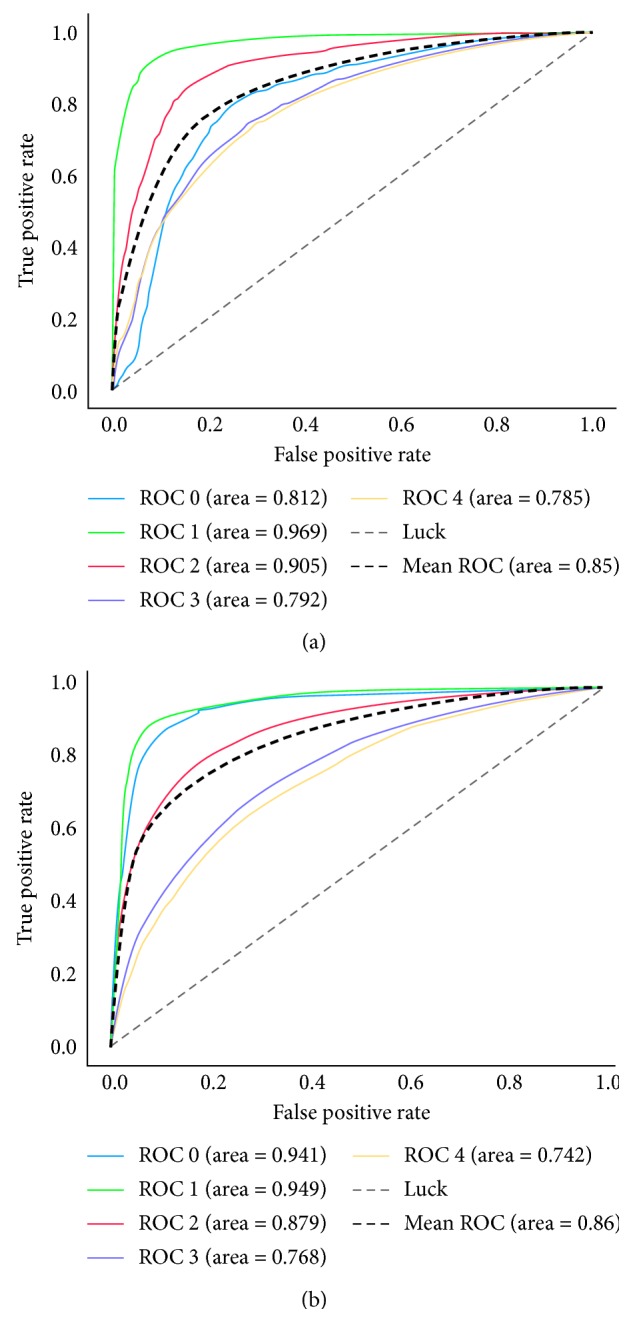
(a) ROC of support vector machine; (b) ROC of random forest.

The highest accuracy rate obtained on the feature classification method SVM is 83.46%, and the highest *F*1-score is 0.8334. The specific results are shown in Table 2. The ROC (receiver operating characteristic) curve is drawn for the trained SVM classifier, as shown in Figure 1(a). The ROC curve in the graph shows the results for each of the 5-fold cross validation. The thicker broken line shows the average ROC with an average AUC (area under the curve) of 0.85 [11].

### 2.2. Random Forest Evaluation

The highest accuracy obtained on RF is 84.50%, and the highest *F*1-score is 0.8334. The specific results are shown in Table 3. The ROC curve is plotted against the trained RF classifier, as shown in Figure 1(b). The ROC curve in the graph shows the results for each of the 5-fold cross validations. The thicker dashed line shows the average ROC curve with an average AUC of 0.86 [11].

Comparing Figures 1(a) and 1(b), and Tables 2 and 3, the performance of SVM and RF are similar. Therefore, the accuracy of feature-based method is around 84%.

### 2.3. MKNet Evaluation

The highest accuracy of the MKNet model is 94.70%. The model for obtaining the highest classification accuracy is the model MKNet-C, with a 17-layer network. Next, the experimental results of the models MKNet-A, MKNet-B, and MKNet-C are analyzed in detail.

**Figure 2:**
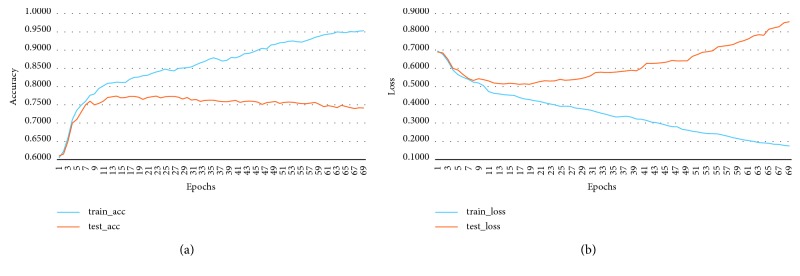
MKNet-A learning curve. (a) Accuracy; (b) loss.

MKNet-A contains only one convolution block conv1. The convolution kernel size of this convolution block is 9∗9, which is replaced by four 3∗3 convolution kernels, to reduce the computing complexity [12]. The learning curve of MKNet-A is shown in Figure 2 [13]. The highest accuracy rate of the model is about 76%, which is quite different from the expected value. A convolution block is added to MKNet-B on the basis of A, from the 5th layer of MKNet-A to the 9th layer of MKNet-B.

**Figure 3:**
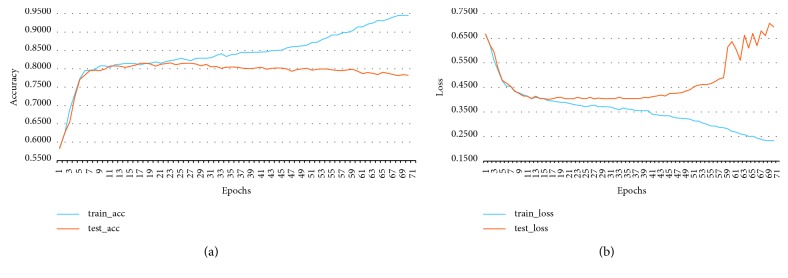
MKNet-B learning curve. (a) Accuracy; (b) loss.

The learning curve of MKNet-B is shown in Figure 3. The accuracy rate of MKNet-B is about 80%, which is 5% higher than that of MKNet-A, but it is still low. Therefore, using MKNet-C with one more convolution block conv3, it is expected to improve the accuracy [14]. And add some dropout layer based on MKNet-B to prevent the overfitting problem caused by the increase of model complexity [15].

**Figure 4:**
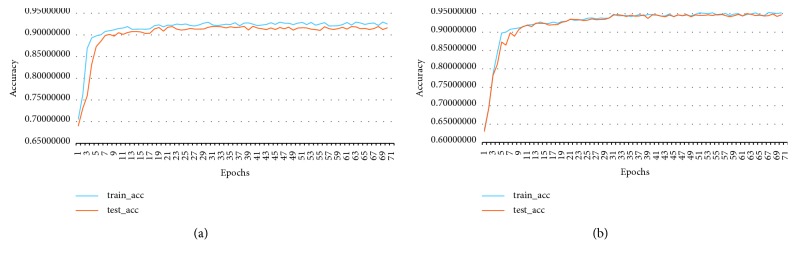
MKNet-C learning curve. (a) No data enhancement; (b) with data enhancement.

The learning curve of MKNet-C is shown in Figure 4(a). MKNet-C has one more convolution block on the basis of MKNet-B and adds 1∗1 convolution layer to each convolution block to increase the fully connected layer fc2. Therefore, the 9-layer MKNet-B becomes the 17-layer MKNet-C.

MKNet-C has an 11% improvement in accuracy over MKNet-B. However, there is still a slight overfitting of the model, so MKNet-C continues to be optimized and trained, using the image enhancement function to increase the original data set [16]. The learning curve of MKNet-C is shown in Figure 4(b). The data enhancement parameter is set to width_shift_range = 0.2, and the original image is randomly moved 20% in the horizontal direction to increase the data set size [16]. The data set after data enhancement is input into MKNet-C for training, which solves the overfitting problem of MKNet-C and finally obtains the accuracy of 94.70%.

The ROC curve is drawn for the trained MKNet-C (with data enhancement), as shown in Figure 6(a), AUC = 0.95, which achieves a good classification performance.

### 2.4. MKRNN Evaluation

**Figure 5:**
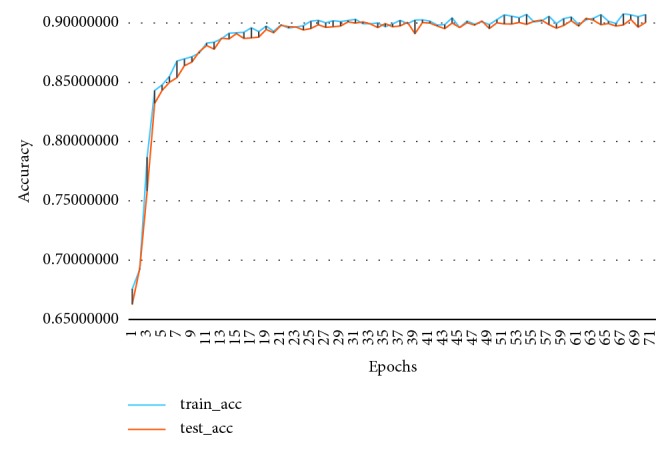
MKRNN learning curve.

The highest accuracy of MKRNN is 90.30%, and the learning curve is shown in Figure 5. The model converges quickly and the highest accuracy rate is 90.30%, AUC = 0.91.

**Figure 6:**
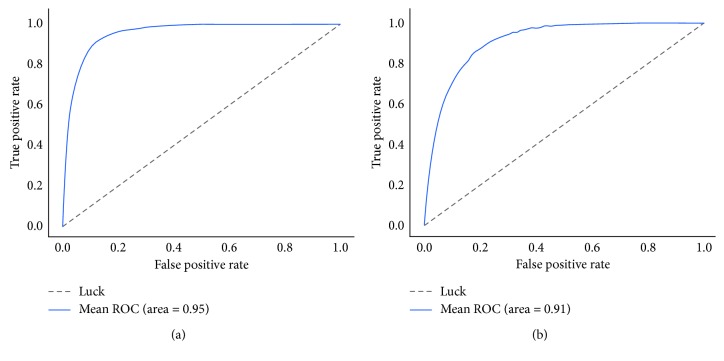
MKNet-B learning curve. (a) ROC of MKNet-C; (b) ROC of MKRNN.

The AUC value of MKRNN is lower than that of MKNet-C, but higher than that of the traditional feature-based classification method [11]. As shown in Figure 6, the accuracy of MKNet is significantly higher than that of MKRNN. The AUC of MKNet is larger than that of MKRNN. Therefore, MKNet shows better performance in the classification of FHR data [11].

## 3. Discussion

The following is a summary of the work of the research topic:

1. Research the current research status and research methods for classification of fetal heart monitoring, including support vector machines, random forests in traditional feature classification methods, CNN in neural network algorithms, and recurrent neural networks. The explanation of the advantages and disadvantages of each research method is given. Focusing on the CNN, this paper analyzes the guiding significance of the CNN on the classification of fetal heart monitoring and proposes the research ideas of this article, pointing out that the neural network is innovative and feasible for fetal heart monitoring classification.
2. Preprocessing the fetal heart monitoring data, sequentially removing the fetal heart rate data for sample deletion with a high proportion of deletions, consecutive missing values, linear interpolation, smooth denoising, and then adjusting the data structure to obtain three different types of data format.
3. Set the control group and the experimental group, and design different algorithms according to different data formats. The support vector machine algorithm and random forest algorithm are used to classify the fetal heart features data. MKNet algorithm and MKRNN algorithm are used to design the classification model of fetal heart rate data. The principle and design process of each model are introduced in detail, and the advantages and disadvantages of the above methods are analyzed.
4. Make comparative analysis of the experimental results of different classification models, optimize and improve the optimal CNN model, prove that the neural network is innovative and feasible for fetal heart rate monitoring classification, and finally get MKNet as the best fetal heart monitoring classification prediction model.

In this chapter, we compare and analyze the experimental results of the four classification models designed and implemented, and conclude that the neural network algorithm is more advantageous in the classification of fetal heart monitoring data. In the neural network model, MKNet shows better performance than MKRNN. According to the experimental results, the traditional feature classification method is difficult to continue to improve after the accuracy rate reaches 84%. This is closely related to the quality of the training data set. The classification method based on neural network, whether it is MKNet or MKRNN, has reached at least 90% accuracy. By increasing the convolution block of MKNet-A, the classification accuracy can be gradually increased. The MKNet-C is the most complex in implementation, and it also obtains optimal prediction results that are superior to all the above models. The accuracy of the feature-based classification method largely depends on the quality of the training data set, but it is difficult to form a high-quality data set by the manually labeled features. A neural network-based classification method can process data by a data preprocessing algorithm to form a high-quality data set. From the results of comprehensive experiments, MKNet outperforms feature-based classification in the difficulty of data set collection and it also outperforms traditional feature classification in classification performance. In summary, this paper trains MKNet as the final model, which has obvious advantages both from the rigorousness of the algorithm design principle and the comparison of the prediction results.

## 4. Materials and Methods

This section introduces data collection, data preprocessing, and model design.

### 4.1. Data Collection

The FHR data set used in this paper was collected by the micro fetal heart monitor, which was from more than 20 hospitals' gynecology across the country. The patient uses a fetal heart rate monitor to perform fetal heart monitoring and uploads the monitoring graphic to the system. The qualified obstetrician remotely interprets the data through a networked computer and a smartphone.

In terms of software, the terminal control device judges the data initially collected by the fetal heart rate instrument to determine the start time of collecting the fetal heart data, record the fetal heart data and the fetal movement time point, and generate a corresponding detection report. On the hardware side, the fetal heart rate Doppler signal received by the probe of the fetal heart monitor is preamplified, demodulated, amplified, and filtered by the posterior pole and sent to the microprocessor for A/D conversion, and then a series of complex calculations are carried out to get the frequency of fetal heartbeats per minute [17].

The label of each sample is reinterpreted by a specially formed panel of experts consisting of three professional authoritative experts. The sample label is based on the consensus of the experts.

### 4.2. Data Preprocessing

The original data are divided into two parts: one is the FHR data collected by the fetal heart monitor, and the other is the medical feature marked by the doctor based on the fetal heart rate curve. In total, there are 24,360 samples. The FHR feature is stored as data fields and used as control group data. This article selects 60% of the data samples as a training set, 20% as a test set, and 20% as a validation set.

**Figure 7:**
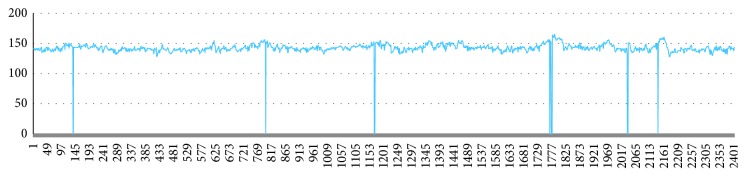
A small amount of missing data.

**Figure 8:**
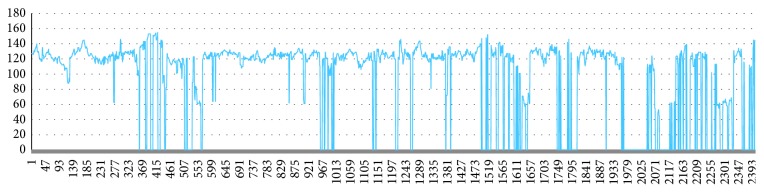
A large amount of missing data.

**Figure 9:**
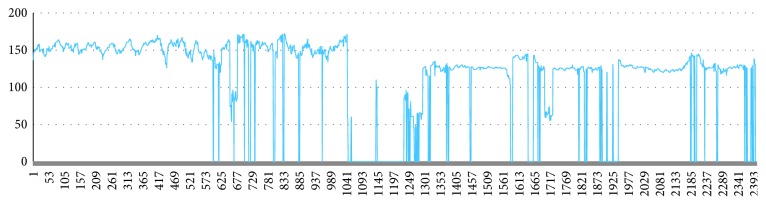
Missing data consecutively.

A part of the original data has a large amount of data missing, and the missing data exist in the raw data as a zero value. The sample in Figure 7 is missing in a small amount and can be filled by a simple missing value padding method. The sample in Figure 8 has a large number of zeros. There are multiple long consecutive missing lines in Figure 9. The missing interval may lose the acceleration or deceleration characteristics.

**Figure 10:**
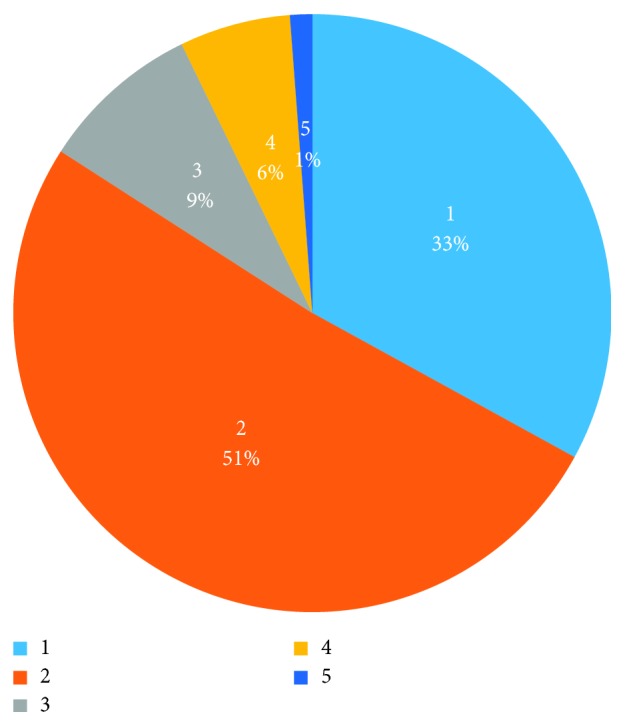
Statistics of the missing data.

The statistics of the missing data of the raw data are shown in Figure 10. 1.2% of the data has a missing of about 1000 points, and 14.7% of the data has a missing of 200 to 500 points. 51.1% of the data is relatively complete; there are only about 100 data points missing and 33% of the data is not missing. Moreover, due to environmental factors such as fetal heart rate monitoring and maternal physical factors, the measured FHR data may be unstable, which may seriously interfere with the results. This unstable data are called dirty data.

In this paper, the methods used for the data preprocessing are as follows:

1. The missing heart value of the FHR signal curve is counted, and the sample whose ratio is greater than the 10 s is rejected [18].
2. Breakpoint detection is performed on the FHR signal curve, and samples with consecutive breakpoints exceeding 30 s are rejected.
3. Linear interpolation missing value repair for FHR signal curve [19].
4. For FHR signal curve for noise reduction, heart rate five times lower than 10 bpm (beats per minute) is regarded as the stable heart rate. Whenever the difference between neighboring heart rates is higher than 25 bpm, the sample will be replaced by a linear interpolation between the previous heart rate and the new stable heart rate.

**Figure 11:**
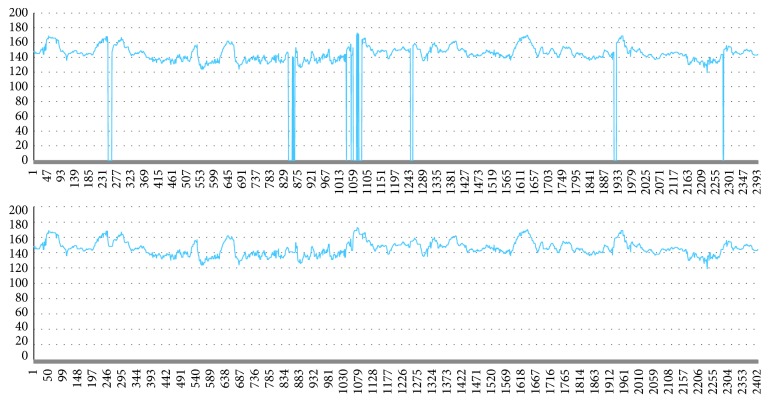
FHR curve after linear interpolation.

Figure 11 shows the curve after a small number of missing samples are filled by linear interpolation, in which the upper image is the original curve and the lower image is the corrected FHR curve.

From the figure, we can significantly see data still exist small jagged unstable fetal heart rate signal, largely by instrument measurement error result in. In order to reduce jagged graphic image of the trend, the use of filters smoothes the curve. Ignoring local small curve change on overall curve trend. This paper for FHR data used Savitzky–Golay filter, which is a digital filter [20].

**Figure 12:**
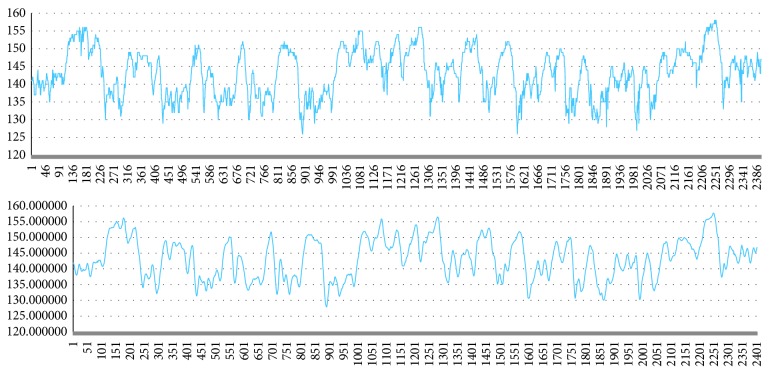
The data after noise reduction.

Figure 12 shows the FHR curve after processed by the smoothing algorithm. It can be observed that the local fine jagged unstable signal is replaced by a smooth curve while retaining the original curve trend.

**Figure 13:**
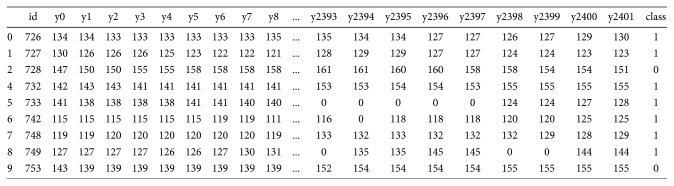
Time series data.

Each sample was a continuous FHR monitoring sample for 20 minutes; a total of 2400 times were recorded in the time of 20 minutes [21]. Thus, each sample contains 2400 fetal heart rate values. The raw FHR data are in json format storage; Figure 13 shows the fetal heart rate time series data extracted from the json format data, which will be used as input data for the circulating neural network.

**Figure 14:**
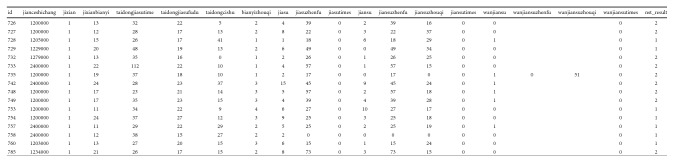
Fetal monitoring data field.

In the raw data, there are medical features manually calibrated according to the FHR curve; these feature fields will be used as input data for feature-based classification methods, as shown in Figure 14.

The 11 fields of the 24360 samples in the experimental data set are used as input for the feature-based classification method. Some of the input data samples are shown in Table 4.

The raw FHR data exist in the form of continuous data points, and the result presented to the doctor is the fetal heart monitoring image, which is the FHR curve transformed from the original continuous data points. Expressed in the form of curves, it is more intuitive and more convenient to see the characteristics of curves and the medical information represented by these characteristics. In order to unify the size of the image data, the rate of fetal heart rate below 80 beats/minute is regulated to 80 beats/minute, and the heart beat rate of more than 200 beats/minute is standardized to 200 beats/minute. The resulting image has 120 pixels on the vertical axis and 2400 pixels on the horizontal axis, so the image size is 120∗2000 pixels. The fetal heart monitoring image will serve as input data to the convolution neural network.

We performed a format conversion of the fetal heart monitoring data, converting the original fetal heart rate data to continuous data containing only the fetal heart rate values, and displaying images of the fetal heart rate curve. The database fields were extracted and the fields with categorical value were selected. Therefore, after data preprocessing, a total of three types of data are obtained. Different algorithms will be used to model different data types.

1. Continuous fetal heart rate values represent changes in fetal heart rate as a function of time, which is a time series data. The model MKRNN will be used to train the model based on the recurrent neural network.
2. The FHR curve represents the changing trend of the fetal heart rate throughout the monitoring time and is the image data. Model training based on the convolution neural network algorithm MKNet will be used.
3. The FHR feature field, which represents the medical characteristic value analyzed from the fetal heart rate curve [22]. Support vector machines and random forests will be used for model training [9].

This article will focus on these three different types of data features: (1) and (2) groups for the experiment and (3) the control group, respectively, for the model design and experiment, and the experimental results were compared.

### 4.3. MKNet Based on Convolution Neural Network

This paper designs a convolution neural network model named “MKNet”; firstly, the important parameters involved in MKNet are introduced, as is shown in Table 5. The Keras deep learning framework is used in this experiment. TensorFlow is served as backend.

**Figure 15:**
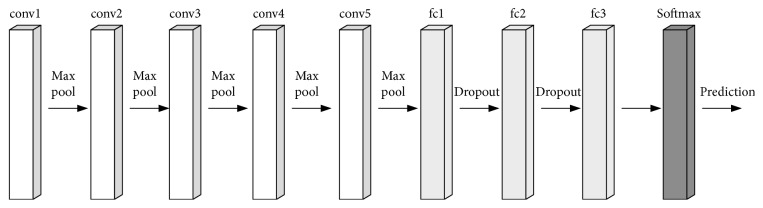
VGG network structure.

Figure 15 is the simplification of the VGG (Visual Geometry Group) network structure. The VGG network is mainly composed of five convolution blocks, three full connection layers, and one softmax layer [23].

Each convolution block contains three convolution layers, which are characterized by continuous convolution layers and large calculations. This article will use the VGG network structure as a reference, starting from a convolution block, gradually build a convolution neural network suitable for fetal heart monitoring image classification. The input data of MKNet in this paper are gray image data, with a size of 120∗2400 pixels.

**Figure 16:**
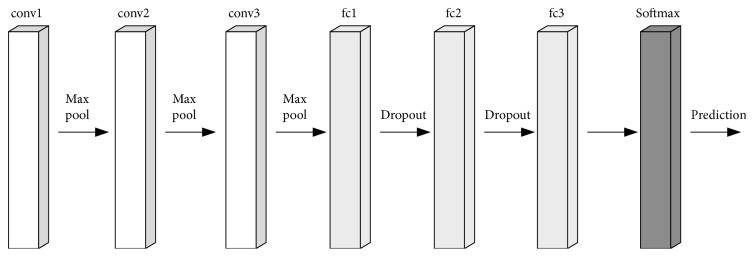
MKNet structure.

In this paper, the 3∗3 filter in the VGG network structure can make the network depth deeper and play the role of implicit regularization, which can make the network converge with only a few iterations [24]. And 2∗2 maximum pooling is employed. When the network is initialized, bad weights have a greater impact on network training. The shallow network is trained in a random manner. When training other network layers, the weights of the shallow network are taken as the initial values of other networks [25]. The following describes the basic structure of the convolution neural network designed in this paper, as shown in Figure 16:

1. The conv1+fc1+softmax of the solid line in Figure 16 is the basic structure of MKNet, which is called network MKNet-A. MKNet-A has one convolution block conv1. The convolution block contains four convolution layers and one largest pooling layer, plus a full connection layer, totaling seven layers. After training MKNet-A, MKNet-A is adjusted based on the experimental results.
2. If the experimental result of MKNet-A is not ideal, add a convolution block conv2 based on MKNet-A, conv2 also contains 4 convolution layers and 1 largest pooling layer, which is called MKNet-B; thus, MKNet-B has 12 layers in total.
3. MKNet-C can also be designed on the basis of MKNet-B, and conv3 convolutional blocks can be added on the basis of convolution blocks conv1 and conv2. This results in a total of 17 layers of MKNet-C. It depends on the situation whether to increase the 1 × 1 convolutional layer and the fully connected layers fc2 and fc3.

In the neural network training process, one problem often encountered is overfitting. To improve the ability to generalize, one way is to use more training data, as the size of the data set is limited in this paper, the data enhancement can be used to solve this problem. In Keras, data enhancements can be implemented with function keras.preprocessing.image.ImageGenerator, which can set random transformations to be applied to data during training [26].  Table 6 shows the parameters used in data enhancement. Because the FHR curve in this article represents the corresponding medical meaning, it cannot be reversed up or down or left or right without violating its original meaning. The feasible method is to carry out small-scale horizontal random movement of the image so that the key information of the image is not lost.

### 4.4. MKRNN Based on Recurrent Neural Network

In this paper, we design the recurrent neural network model as MKRNN [27]. The important parameters involved are shown in Table 7. The input layer of the LSTM (long short-term memory) is specified by the input_shape parameter on the first hidden layer of the neural network. The LSTM layer must prespecify the shape of the input, and the input to each LSTM layer is three-dimensional. You need to convert the original time series data into three dimensions. The three dimensions that need to be entered are the sample size, time step, and feature [25]. (1) Number of samples: A sequence is a sample, and the number of samples is greater than 1 by default. (2) Time step: A time step represents one observation point in the sample. (3) Features: A feature is observed in a time step, equivalent to each column in the data. Based on the above analysis and design, we have determined the final model of the recurrent neural network and described the recurrent neural network model according to the training process.

### 4.5. Feature-Based Method

The feature-based classification method is build as control group in this paper, SVM and RF are chosen as the classifier.

After the search of the grid search algorithm, the combination of the optimal hyperparameters of the SVM is {‘kernel': ‘rbf', ‘C': 1000, ‘gamma': 0.0001}, all the other hyperparameters are set in default value.

## 5. Conclusions

The following is a summary of the work of the research topic:

1. Research the current research status and research methods for classification of fetal heart monitoring, including support vector machines, random forests in traditional feature classification methods, convolutional neural networks in neural network algorithms, and recurrent neural networks. The explanation of the advantages and disadvantages of each research method is given. Focusing on the convolutional neural network, this paper analyzes the guiding significance of the convolutional neural network on the classification of fetal heart monitoring, and proposes the research ideas of this article, pointing out that the neural network is innovative and feasible for fetal heart monitoring classification.
2. Preprocessing the fetal heart monitoring data, sequentially removing the fetal heart rate data for sample deletion with a high proportion of deletions, consecutive missing values, linear interpolation, smooth denoising, and then adjusting the data structure to obtain three different types of data format.
3. Set the control group and the experimental group, and design different algorithms according to different data formats. The support vector machine algorithm and random forest algorithm are used to classify the fetal heart features data. MKNet algorithm and MK-RNN algorithm are used to design the classification model of fetal heart rate data. The principle and design process of each model are introduced in detail, and the advantages and disadvantages of the above methods are analyzed.
4. Make comparative analysis of the experimental results of different classification models, optimize and improve the optimal convolutional neural network model, prove that the neural network is innovative and feasible for fetal heart rate monitoring classification, and finally get MKNet as the best fetal heart monitoring classification prediction model.

## Figures and Tables

**Table 1:** Comparison of performance of different models.

Parameter	SVM	RF	MKNet	MKRNN
Training time	118.90 s	14.35 s	1330 s	350 s
Precision	83.45	84.49	94.71	90.33
Recall	83.46	84.50	94.68	90.28
Accuracy	83.46	84.50	94.70	90.30

**Table 2:** SVM model classification results.

Label	Precision	Recall	*F*1-score
0	0.8364	0.8890	0.8619
1	0.8318	0.7594	0.7939
Avg/total	0.8345	0.8346	0.8334

**Table 3:** SVM model classification results.

Label	Precision	Recall	*F*1-score
0	0.8364	0.8890	0.8619
1	0.8318	0.7594	0.7939
Avg/total	0.8345	0.8346	0.8334

**Table 4:** Fetal heart rate features.

Baseline	Acceleration time	Acceleration magnitude	Frequency	Period	Acceleration	Acceleration amplitude	Deceleration	Deceleration period	Result
13	32	22	5	2	4	39	2	39	2
12	28	17	13	2	8	22	3	22	2
15	26	17	41	1	1	18	6	18	1
20	48	19	13	2	6	49	0	49	1

**Table 5:** Convolution neural network parameters.

Parameter	Description	Default
Filters	The number of filters in the convolution operation	8, 16, 32
kernel_size	The size of the convolution kernel	(3, 3), (5, 5)
Strides	Convolutional step size	(4,4)
Activation	Activation function	relu, adam
pool_size	Pool size	(2,2)
Padding	Whether zero padding	Same, valid
drop_out	The proportion of nonworking neurons	0.25
Optimizier	Optimization function	rmsprop, adagrad
Epochs	The number of complete training samples of the data set	50, 100
batch_size	The number of training samples per iteration	32, 64, 128
data_augmentation	Whether to use data enhancement	True

**Table 6:** Data enhancement parameters.

Parameter	Description	Default
rotation_range	Specifies the angle for randomly selecting pictures	0 to 180°
width_shift_range	Specifies the proportion of random shift horizontally	Ratio between 0 and 1
height_shift_range	Specifies the proportion of random shift vertically	Ratio between 0 and 1
shear_range	The degree of shear transformation	Ratio between 0 and 1
zoom_range	Random zoom	Ratio between 0 and 1
horizontal_flip	Randomly flips the picture horizontally	False
vertical_flip	Randomly flips the picture horizontally	False

**Table 7:** Recurrent neural network parameters.

Parameter	Description	Default
units	The number of RNN units or an output dimension	Positive integer
Activation	Activation function name	relu, adam, tanh
drop_out	The input linear transformation of the neuron opening ratio	Ratio between 0 and 1
recurrent_dropout	The neuron disconnection ratio of the cyclic state linear transformation	Ratio between 0 and 1
return_sequences	The return of the last output of the output sequence	True, False
go_backwards	Reverses the input sequence and returns the reversed sequence	True, False
Stateful	The final state of a sample being used as the initial state for the next batch	True, False
input_dim	When using the layer as the first layer of the model, specify the value	Positive integer
input_length	The length of the input sequence, or specify this parameter	Positive integer
merge_mode	Combination of forward and backward RNN outputs	sum, mul, none

## Data Availability

The fetal heart monitoring data used to support the findings of this study are currently under embargo, while the research findings are commercialized. Requests for data (6/12 months after publication of this article) will be considered by the corresponding author.
